# Functional Evolving Patterns of Cortical Networks in Progression of Alzheimer's Disease: A Graph-Based Resting-State fMRI Study

**DOI:** 10.1155/2020/7839536

**Published:** 2020-06-29

**Authors:** Wei Li, Wen Wen, Xi Chen, BingJie Ni, Xuefeng Lin, Wenliang Fan, The Alzheimer's Disease Neuroimaging Initiative

**Affiliations:** ^1^The School of Artificial Intelligence and Automation, Huazhong University of Science and Technology, Wuhan 430074, China; ^2^Image Processing and Intelligent Control Key Laboratory of Education Ministry of China, Huazhong University of Science and Technology, Wuhan 430074, China; ^3^Department of Radiology, Union Hospital, Tongji Medical College, Huazhong University of Science and Technology, Wuhan 430022, China; ^4^Hubei Province Key Laboratory of Molecular Imaging, Wuhan 430022, China; ^5^Alzheimer's Disease Neuroimaging Initiative Study, USA

## Abstract

AD is a common chronic progressive neurodegenerative disorder. However, the understanding of the dynamic longitudinal change of the brain in the progression of AD is still rough and sometimes conflicting. This paper analyzed the brain networks of healthy people and patients at different stages (EMCI, LMCI, and AD). The results showed that in global network properties, most differences only existed between healthy people and patients, and few were discovered between patients at different stages. However, nearly all subnetwork properties showed significant differences between patients at different stages. Moreover, the most interesting result was that we found two different functional evolving patterns of cortical networks in progression of AD, named ‘temperature inversion' and “monotonous decline,” but not the same monotonous decline trend as the external functional assessment observed in the course of disease progression. We suppose that those subnetworks, showing the same functional evolving pattern in AD progression, may have something the same in work mechanism in nature. And the subnetworks with ‘temperature inversion' evolving pattern may play a special role in the development of AD.

## 1. Introduction

The pathogenesis of Alzheimer's disease (AD) is concealed and affects both the brain structure and function connections [1]. It can be divided into three main phases: preclinical, mild cognitive impairment (MCI) which can be further divided into the early stage of MCI (EMCI) and the later stage of MCI (LMCI), and dementia [2]. It is reported that about 10–15% individuals with MCI tend to progress to AD per year [3]. Although some studies claimed that acetylcholinesterase inhibitors may lower the rate of this progression, [4, 5] some did not support this conclusion [6, 7]. The disagreement prompted us to further study how the brain changes during the different stages of the disease.

Resting-state brain subnetworks showed the pathological features of AD especially DMN [8, 9], and the subnetworks reflected interactions with each other such as competitive relationship [10], regulatory role, [11] or transition player [12]. Therefore, it is crucial that we should not only notice the changes in the whole brain but also focus on the alterations in each subnetwork and the intra- or internetwork connection to discover the pathogenesis of AD. Using the graph theory method, there were some differences between normal controls and patients, such as a lower value of the small-word attribute and clustering coefficient in AD [13], significant decrease of clustering coefficient, and local efficiency in the limbic network of MCI [14]. Due to the samples' heterogeneity of the different methods, the results are inconsistent and even contradictory. Furthermore, the change patterns of each network properties in the process of EMCI to LMCI and AD and how the inter- and intrasubnetworks are altered are still unknown.

Based on resting-state functional magnetic resonance imaging (fMRI), we used the graph theory method to explore the dynamical change patterns of brain functional network reorganization during the development of AD. We sought to determine the significant brain functional connectivity features to discover the progression pathogenesis and reveal the difference of global network attributes by studying the changes of internal attributes of each resting-state network.

## 2. Subjects and Methods

### 2.1. Information of Subjects

The data used in this study were obtained from the Alzheimer's Disease Neuroimaging Initiative (ADNI) dataset (http://adni.loni.usc.edu/).The primary goal of ADNI has been to test whether serial magnetic resonance imaging (MRI), positron emission tomography (PET), other biological markers, and clinical and neuropsychological assessment can be combined to measure the progression of mild cognitive impairment (MCI) and early Alzheimer's disease (AD). For up-to-date information, see http://www.adni-info.org. Twenty-five patients with AD (age: 75.17 ± 4.08), thirty-three patients with LMCI (age: 74.03 ± 4.65), thirty-seven patients with EMCI (age: 72.96 ± 4.55), and thirty-five age-matched NCs (age: 73.80 ± 5.06) were recruited. The age of subjects in this study ranged from 60 to 90. The demographic information of subjects is shown in Table 1.

### 2.2. Preprocess of Data

The functional MRI images were acquired on 3.0 T Philips scanner. One hundred and forty function volumes were obtained with the parameters (TR/TE = 3000/30, FA = 80°, slice thickness = 3.3 mm, matrix = 64 × 64, and number of slices = 48). To insure magnetization equilibrium, the first ten images of each subject were discarded.

We used Statistical Parametric Mapping (SPM8, https://www.fil.ion.ucl.ac.uk/spm) and Data Processing Assistant for Resting-State fMRI (DPARSF) toolbox to preprocess next. After slice timing and realigning time series, subjects whose head translation exceeded 2 mm or head rotation exceeded 2° were excluded. All images were normalized to the Montreal Neurological Institute (MNI) template for consistency. Then, we smoothed the images with a standard 4 × 4 × 4 FWHM kernel and used a bandpass frequency with the range from 0.01 Hz to 0.08 Hz to filter the time courses. Subsequently, to reduce the effects of motion and nonneuronal BOLD fluctuations, the global mean signal, white matter signal, and cerebrospinal fluid signal the covariates which consist of six head motion parameters were removed. Finally, the time series of 90 interest (ROI) based on the anatomical automatic labeled brain (AAL; http://www.cyceron.fr/freeware/) were extracted for each subject.

### 2.3. Construction of Brain Functional Network

The construction of the brain network was performed using MATLAB 2016a and the brain connectivity toolbox (BCT; https://www.nitrc.org/projects/bct).We used Pearson's correlation coefficient to obtain the correlation matrix. Each correlation matrix was further divided into the binary matrix with a fixed sparsity value *S*, which was defined as the ratio of the sum of existing edges divided by the most possible number of edges in a network. In order to study the network attribute performance, we applied a wide range of sparsity thresholds instead of selecting a single one. We selected the scope of *S* according to the following criterions: (1) the average degree was no less than 2 × log(*N*) and (2) the small worldness of the normal control group was no less than 1.1 [15]. These criterions minimized the possible number of fake edges in each network. Eventually, we confirmed *S* with a threshold (0.08, 0.52), and step was 0.01.

In order to characterize the attributes of the brain functional networks, we employed five parameters: the clustering coefficient, efficiency, transitivity, characteristic path length, and small worldness, shown in Table 2. The interpretations and detailed uses can be seen in a previous study [16]. Moreover, we calculated the area under the curve (AUC) over a range of density instead of selecting a single sparsity threshold for each binary network, which offered a summarized scalar for the network topological attributes and avoided the influence of selecting a single threshold [17, 18]. The method has been widely used in many studies [19–21].

Correspondingly, we divided 90 regions into five RSN which included the default mode network (DMN), attention network (ATT), subcortical network (SUB), auditory network (AUD), visual network (VIS), and sensorimotor network (SEN) [22, 23]. We also calculated the global attributes of the RSNs whose connectivity matrix was the functional connectivity of paired regions belonging to the RSNs *R*_*n*_ = {*r*|*r*_*ij*_^*n*^ = corr(*r*_*i*_, *r*_*j*_), *n* = 1 ⋯ ⋯5, *i*, *j* ∈ *n*}, and binary networks also consisted of the regions belonging to the RSNs *B*_*n*_ = *b*_*ij*_^*n*^, *n* = 1 ⋯ 5 [24, 25]. Then, the properties of RSN were calculated and the independent two-sample *t*-test was also used. Afterwards, we calculated the graph theory measurement of the RSNs and the AUC, respectively. For internetwork, the connectivity matrix was composed of functional connectivity of paired regions in any two of the RSNs *C*_*nm*_ = mean(*C*_*nm*_),  *C*_*nm*_ = {*r*|*r*_*ij*_^*nm*^ = *corr*(*r*_*i*_, *r*_*j*_), *n* = 1 ⋯ 5, *i* ∈ *n*, *j* ∈ *m*} [24, 25]. And the two independent sample *t*-test was also used for the FC of the internetwork. The correlation analysis between the properties (both global and local) and MMSE were performed.

### 2.4. Statistical Analysis

To estimate whether there were significant group differences between the metrics, we used a nonparametric permutation test with 10,000 repetitions on the AUC of each network metric [26] between the normal controls and the EMCI/LMCI/AD patients, respectively. After calculating the difference between groups for each network attribute, we randomly reallocated all the values into two groups in order to obtain the empirical distribution of the difference. Then, we used 95 percent of the distribution as confidence in a one-tailed test to find out whether differences between the observed groups could occur casually [19, 27]. In addition, we used a Benjamini Hochberg false discovery rate (FDR) correction method with a significance level of 0.05 to solve the problem of multiple comparisons. For the properties of intra and interfunctional connectivity of RSN, two independent sample *t*-test was used as we mentioned before.

## 3. Result

### 3.1. Global Attributes

**Figure 1:**
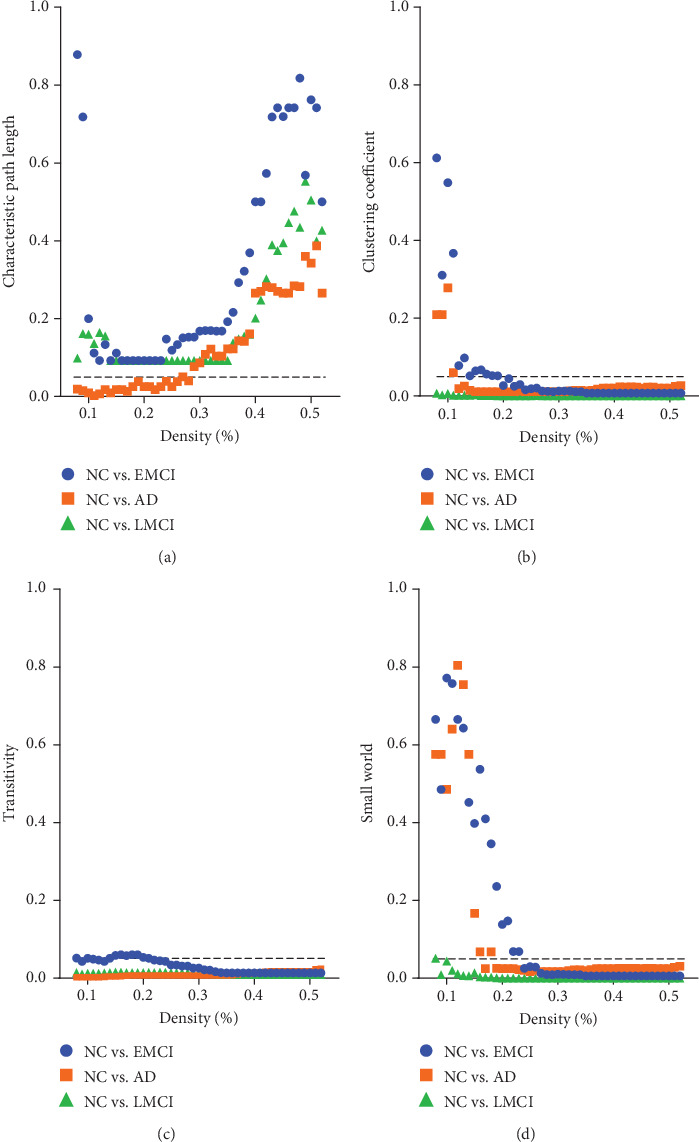
*p* values of each property in different stages.

Based on the functional connectivity adjacency matrix, we calculated the clustering coefficient, characteristic path length, transitivity, efficiency, and small world attribute. We performed a post hoc two-sample *t*-test on any two groups of NC, EMCI, LMCI, and AD. In the results, only the NC group showed a significant difference, respectively, with the EMCI, LMCI, and AD groups on the clustering coefficient, characteristic path length, transfer coefficient, and the small world attribute. Other group combinations showed no difference on any global network properties.  Figure 1 showed the *p* value (FDR correction) of the *t*-test of those four brain network attributes showing a significant difference between the NC group and other three groups (EMCI, LMCI, and AD), respectively. The NC group showed significant differences with EMCI on the clustering coefficient, transitivity, and small world attribute at most of various brain network densities. Compared with LMCI, the NC group has a significant difference at most brain network densities on the characteristic path length, clustering coefficient, transitivity, and small world attribute. In the NC group vs. the AD group, there also are differences on the characteristic path length, clustering coefficient, transfer coefficient, and small world attribute.

**Figure 2:**
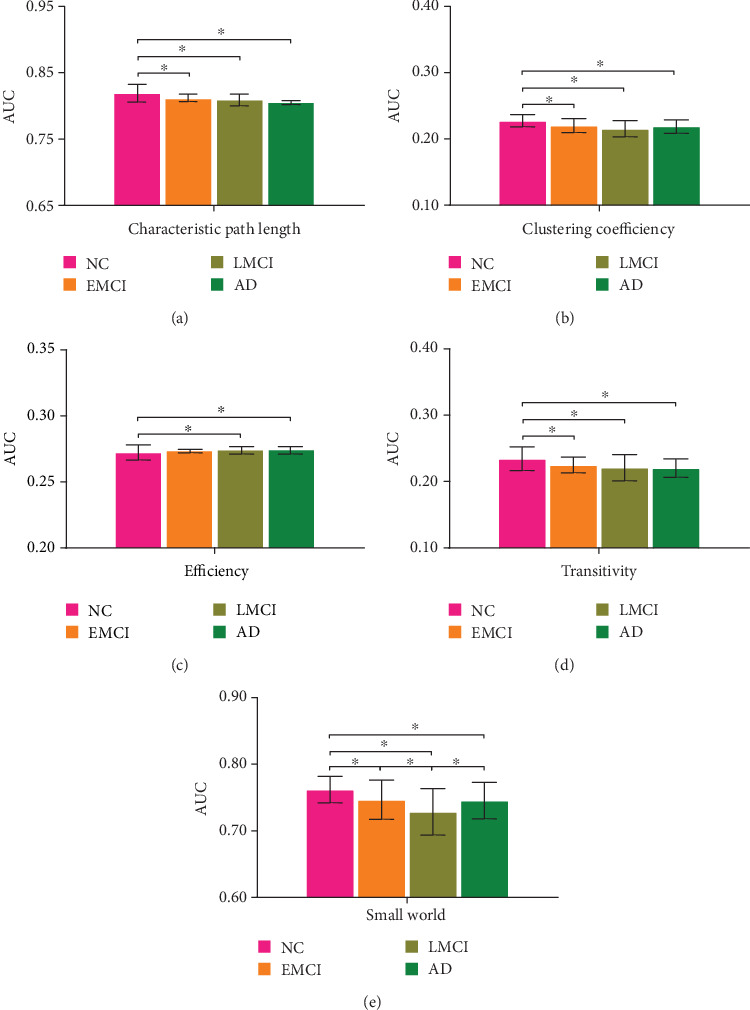
AUC of each property in different stages.

Since there is no unified opinion on the demarcation of brain network density, we used the global attribute AUC to remove the effect of density selection on network attributes analysis, as well as the characteristic path length, clustering coefficients, efficiency, transitivity, and small world attribute. We found that on the characteristic path length, transitivity, and clustering coefficient, only the NC group showed significant differences compared with the EMCI, LMCI, and AD groups. In efficiency, only the NC group compared with LMCI and AD was different, while groups from other stages have no difference with each other. In the small world attribute, all group combinations showed significant differences except EMCI vs. AD. It can be found that in the progression from NC to AD via EMCI and LMCI, the small world attribute showed a U-shaped change curve. There was no difference in other attributes except the small world attribute in EMCI vs. LMCI, which may be because the difference of these attributes between the two groups was to reach a significant level. For EMCI vs. LMCI and LMCI vs. AD, only the small-world attribute was significantly different and there was no difference on other attributes. As can be seen in Figure 2, in addition to the small world attribute and clustering coefficient which showed U-shaped changes in the disease progression, the other three attributes matched continuous monotonous decreasing trend.

### 3.2. Functional Connectivity

**Figure 3:**
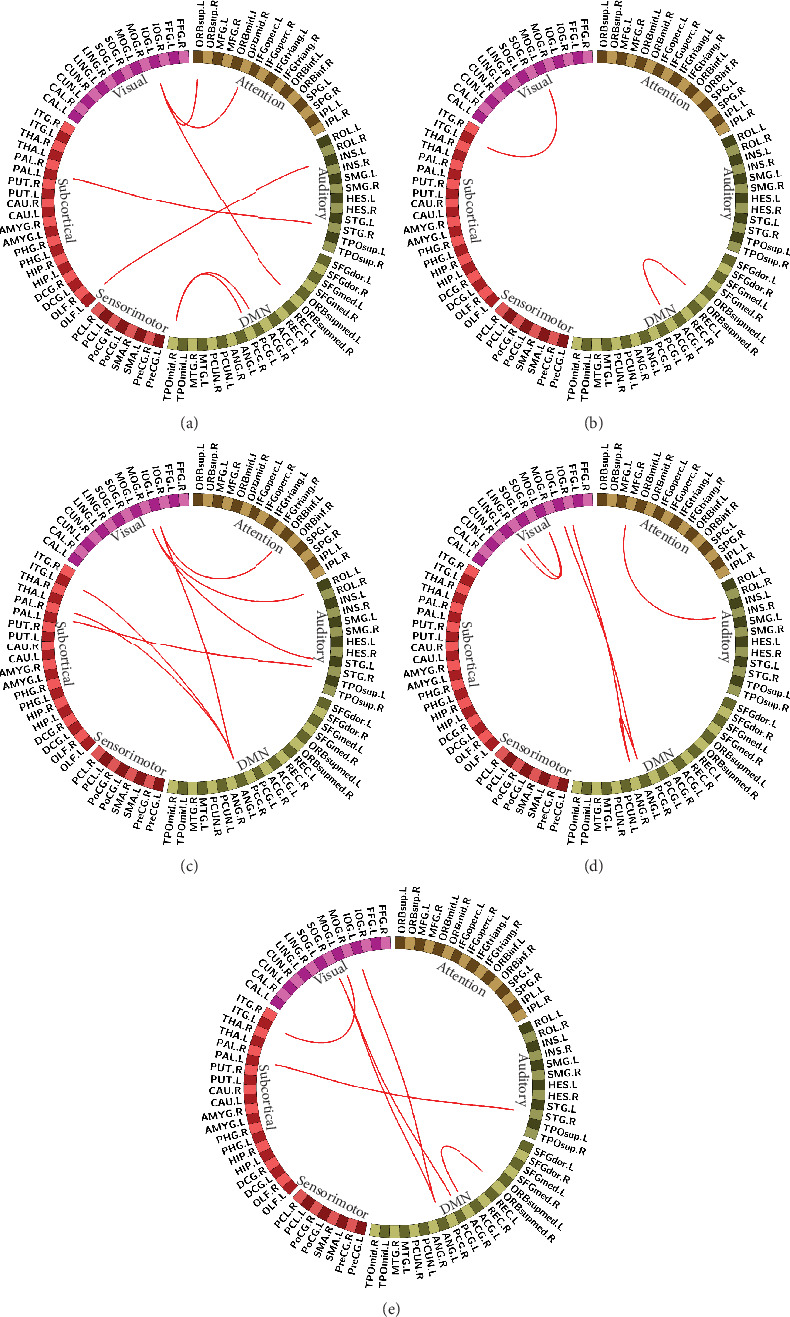
Functional connectivity with significant differences in stages. (a) NC compared with EMCI. (b) EMCI compared with LMCI. (c) LMCI compared with AD. (d) NC compared with AD. (e) EMCI compared with AD.

Post hoc two-sample *t*-test (FDR corrected, 1000 permutation) was performed on the same region connection of the brain network between any two groups of NC, EMCI, LMCI, and AD. Eventually, we could gain pairs of brain regions with significant differences in functional connectivity between groups. As we can see in Figure 3, seven functional links showed significant differences between the NC group and EMCI group, including IOG. L and ORBsup. L, ORBmid. R, ORBsupmed. R, TPOmid. R and PCG. L, PCG. R, OLF. L and INS. L, and PAL. L and STG.R. Among these links, TPOmid. R, PCG. L, and PCG. R belong to the DMN network, and other link brain regions belong to different subnetworks. There were significant differences between EMCI and LMCI in only two edges of the brain network, MOG. L and ITG. L, ORBsupmed. R and ACG.R. For the LMCI group and AD group, seven links of brain regions were found to be significantly different, IOG. L and ORBinf. L, ANG. L, PAL. L and STG. R, ANG. L and ITG. L, ROL. L and MOG. R, ANG. L and PAL. R, and MOG. R and STG.L. For these links, brain region pairs all belonged to different subnetworks apart. Further, we performed a Pearson correlation analysis on the weights of these edges with significant differences and the MMSE scores of the corresponding groups, respectively, and found that the link weight between the MOG. L and ITG. L had a significant correlation with MMSE scores (*r* = −0.43, *p* = 0.008) in the EMCI group, so did the link weight between the ANG. L and SOG. R (*r* = 0.41, *p* = 0.012). It was also observed that the link weight between IOG. R and ANG. R was significantly associated with AD group's MMSE scores (*r* = 0.41, *p* = 0.043).

**Figure 4:**
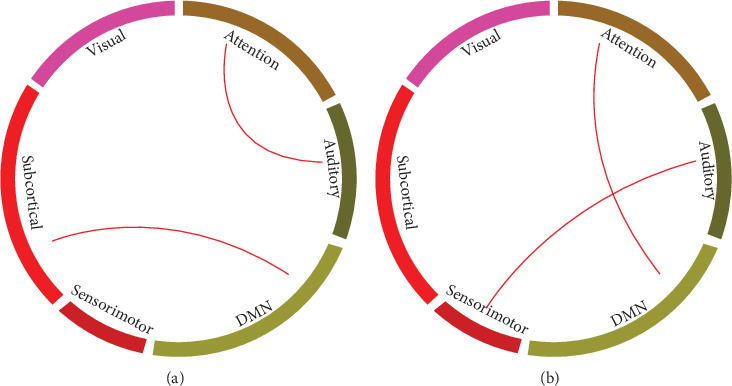
Significant difference of function connectivity in the intersubnetwork. (a) NC compared with EMCI. (b) NC compared with AD.

We did a *t*-test on the connections of intrasubnetworks between any two groups of NC, EMCI, LMCI, and AD. As a result, comparing NC with the EMCI group, there was a significant difference only on the link weight of the attention network vs. the auditory network and the default mode network vs. the subcortical network. Comparing the NC group with the AD group, differences were found only on the link weight of the attention network vs. the default mode network and the auditory network vs. the sensorimotor network. As we can see in Figure 4, differences only appeared in the comparison of the NC group with the initial disease stage, EMCI, and the final disease stage, AD.

In addition, there is no significant difference on link weights of the intrasubnetworks between groups. Afterwards, we employed graph theory to explore the topological properties of each subnetwork intending to explore some more differences between groups.

### 3.3. Subnetworks

**Figure 5:**
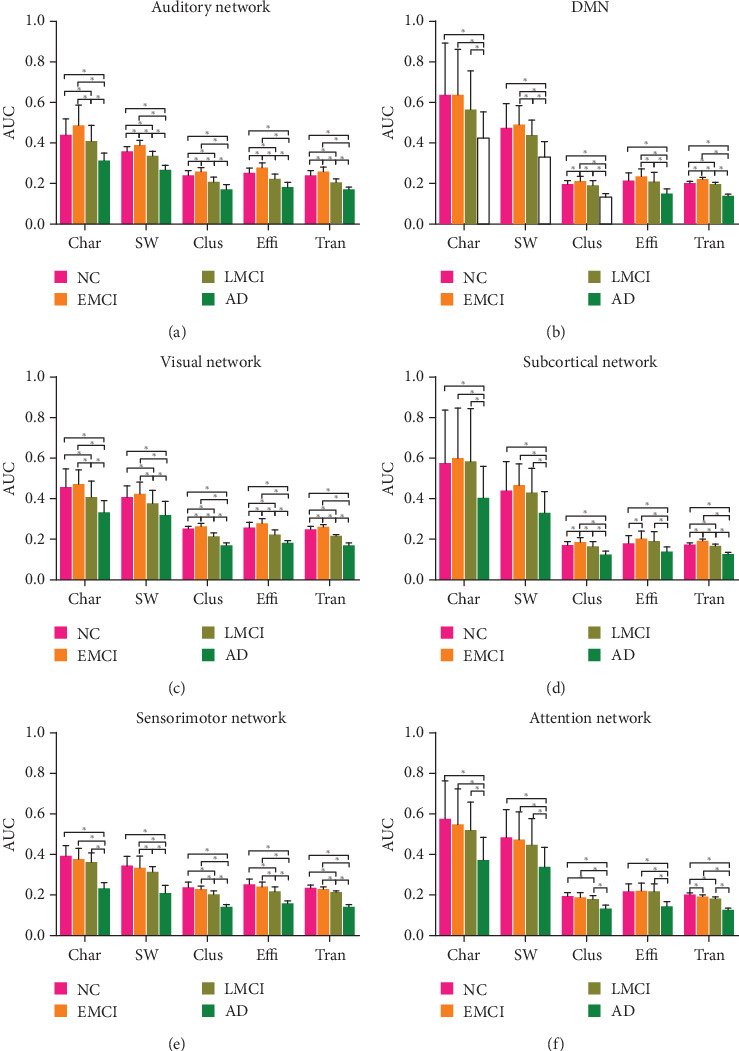
The value of AUC in subnetwork properties. (a) Auditory network. (b) Default mode network. (c) Visual network. (d) Subcortical network. (e) Sensorimotor network. (f) Attention network. ^∗^ represented that there existed a significant difference.

For each subnetwork, we also calculated the AUC of the characteristic path length, small world property, clustering coefficient, global efficiency, and transitivity as we performed a global brain network analysis. At the same time, we also performed two-sample *t*-test on properties of AUC between groups. We find out that the six subnetworks all have differences in the characteristic path length, clustering coefficient, small-world attribute, global efficiency, and transitivity between any two groups, see Figure 5.

In addition, we found that the changing trends of properties of these subnetworks showed two distinct patterns as the disease progress from NC to AD, via EMCI and LMCI.

The properties of the four subnetworks, auditory network, default mode network, visual network, and subcortical network appeared first in an increasing and then decreasing pattern, which is an inverted U-shaped curve as the disease progressed to AD. In the NC-EMCI process, the properties rose and peaked in EMCI. While in the EMCI-LMCI-AD process, all the properties declined, down to the minimum in AD.

On the other hand, the properties of the sensorimotor network and attention network all showed a continuous decline, which we called a “monotonous decline,” in line with our common understanding of this disease, that is, as the disease gets worse, brain areas continue to be attacked, and brain function declines in a monotonous decreasing pattern.

### 3.4. Mini-Mental State Examination Score (MMSE)

MMSE scores of the NC, EMCI, LMCI, and AD group were not related to any of the global attributes (clustering coefficient, small world property, characteristic path length, transitivity, and efficiency) except the characteristic path length in the stage of EMCI and AD (*r* = 0.26, *p* = 0.04). However, in the subnetwork analysis, the MMSE scores were observed to be related to the properties of the DMN in the LMCI stage.

## 4. Discussion

In this study, we used graph theory based on resting-state fMRI data to investigate the alteration of network attributes for the whole brain and each subnetwork following the progresses of AD. Little difference was found in global network attributes between different stages in the progressing process of AD. While in the subnetwork analysis, we found that all five attributes of each subnetwork (the intra- and intersubnetwork functional connectivity) for each progressing stage of AD (EMCI, LMCI, and AD) changed to various degrees, and most of these changes reached statistical significance, especially in DMN and visual network. However, the most interesting and important thing we discovered in this study was that there exist two types of distinct change patterns in the functional brain subnetwork during disease progression in AD.

AD is a progressive disease that spread through axonal pathways to other brain areas during disease progression. It is reported that this propagation is constrained by the underlying white matter brain structural connectome, which may reflect the potential mechanism on how brain network topology shapes neural response to early damage in AD [28]. Our results also support this hypothesis to some extent that we found compared to NC participants; EMCI, LMCI, and AD patients all showed a significant difference in the characteristic path length, clustering coefficient, transitivity, and small worldness, indicating a dynamic reorganization of the brain functional network in the progression of AD.

Thereinto, the small world attribute showed a significant difference between groups of different progressing stages of AD, implying that the information transmission efficiency of the brain network was changed significantly throughout the proceeding of this disease. Meanwhile, an interesting phenomenon is that the small world attribute showed a U-shaped change during the disease progression, but not a monotonous decline. Although small world property is thought to be the optimal topological structure of the brain, the most serious change of this property is not in the final AD stage. We speculate that it is because of the continuous interaction between the progressive disease damage and the underlying brain compensatory mechanisms, which results in the ultimate U-shaped manifestation of the small world parameter.

Except the small world attribute, we did not observe any significant difference on other properties between any two groups of disease stages, including EMCI, LMCI, and AD. Only on the stages of EMCI and AD do the characteristic path length of all global topological attributes showed significant correlation with MMSE (*r* = 0.26, *p* = 0.04) in patients, which indicated their potential application in capturing the progress of AD on the one hand, and that the global metrics for the whole-brain network could only reflect part of the topological alterations on the other hand. This also prompted us to subsequently study on the alterations and interactions of subnetworks.

Base on the previous brain network analysis, we investigated the topological attributes and the interactions of subnetworks. The properties of each subnetwork were significantly altered during the progression from NC to EMCI, LMCI, and AD. The most interesting result was the changing pattern of the subnetworks. The changing patterns of five properties of auditory network, DMN, visual network, and subcortical network all showed an inverted U-shaped curve, which was an ‘inversion' changing pattern from the perspective of disease progression, but not a monotonous decreasing trend in function as we typically supposed. We call this inverted U-shaped change pattern as “temperature inversion phenomenon,” which was derived from meteorological concepts that an inversion is a deviation from the normal change pattern of an atmospheric property with altitude [29]. In the near-surface atmosphere, air temperature increases with height, but does not follow the common rule that air temperature decreases with an increasing altitude. Meanwhile, the peak values of the inverted U-shaped changing curves all located in the same disease progressing stage, EMCI. However, the change patterns of other two subnetworks, the sensorimotor network, and attention network linearly declined with the progression from NC to AD, which we called as “monotonous decline” to represent the monotonous decreasing pattern. We speculate that there may be two different types of subnetworks in the brain that reflect different pathophysiological mechanisms in the progression of AD disease. When the properties of subnetworks change as the disease progresses in a same pattern, we call these subnetworks as “homogeneous evolving subnetworks”, otherwise, we call them “heterogeneous evolving subnetworks.” We suppose that the functional mechanisms of homogeneous evolving subnetworks may have something the same in nature. The specific physiological significance involving homogeneous or heterogeneous evolving subnetworks needs to be further studied in the future.

We suppose that the interactions of these two different types of change patterns of subnetworks finally result in the topological performance of the whole-brain network. Some researchers reported the steady pattern of anticorrelations between the DMN and the task positive network [30–32]. Some studies also reported the negative correlation pattern association to brain development and aging [33, 34]. However, the distinct change patterns with disease progression has never been reported to the best of our knowledge.

Combining our results, we speculated that during the EMCI stages, the brain damage was already widely located in the brain areas, which affected the information transmission ability of the whole-brain network. To minimize the influence, the robustness of the brain triggers the synergy and antagonism among subnetworks for function compensation. When it was in the AD stage, the brain was seriously damaged due to the progression of the disease, which ultimately led the brain network topology to shift to randomization [13, 35]. So the change patterns of the small-worldness metric of the DMN and corresponding homogeneous evolving subnetworks have an ‘inversion' style with that of the whole-brain network. Meanwhile, the characteristic path length and the small worldness of the DMN were significantly negatively correlated with the MMSE scores in the LMCI group, while the efficiency of the DMN was significantly positively correlated with the MMSE scores of LMCI patients, indicating their potential biomarkers for the progress in MCI stages and the importance of researches on the subnetworks such as the DMN in the future.

For the interactions between the subnetworks, we found that compared with the NC stage, the functional connectivity between ATT and AUD and DMN and SUB was significantly decreased in the EMCI, while the functional connectivity between ATT and AUD and AUD and SEN was significantly decreased in the AD stage. The DMN regions were reported to be structurally connected [36] and related to the memory-related cognitive deficits in MCI patients [37]. It was also reported that the DMN exhibits functionally disconnected regions [38] and metabolic disruption [39] as well as atrophy [40] and aberrant amyloid-*β* accumulation [41]. In our edge-wise statistical analysis, we also found the alteration of the functional connectivity mainly is involved with the DMN and visual network during the whole disease progression. These results implied that the interactions between subnetworks may reflect the mechanism of the brain network topological structure changes during progression of diseases. On the other hand, when we focus on the adjacent disease stages, we found that the aberrant functional connectivity almost involved all of the subnetworks except the SEM in the EMCI, while only four edges showed significant changes in the LMCI, which support the hypothesis that there are already some alterations of the brain functional network topological attributes in the EMCI stages as we mentioned before. When compared with NC, almost all subnetworks showed disrupted functional connectivity in the AD stage, which was consistent with the previous studies [42, 43]. Meanwhile, as reported in other studies, some of the altered edges (functional connectivity) were significantly correlated with the MMSE, which suggested the key role it plays in the different stages and helped us characterize the process of the disease. In general, maybe the local-specific subnetworks or edge-wise changes eventually lead to the interesting global network ‘inversion' change pattern.

There are several issues that need to be further addressed. First, in this study, we used cross-sectional data of each stage; some results maybe be more precise if only longitudinal follow-up studies were performed in the future. Second, accumulating evidence suggests the importance of considering the structure and perfusion of the brain. Thus, combining multimodal neuroimaging data will provide more comprehensive insights into the progression of NC to EMCI, LMCI, and AD. Third, there are still some issues about the lack of efficient research methodologies on the subnetwork, which may result in the insufficient analysis result of the subnetwork in the worst cases. The mechanism and foundation of the functional brain network are still unclear, so that using density as a topological criterion for functional brain network may not be appropriate. Moreover, a data-driven topological filtering scheme for the brain network analysis such as the OMST method [37, 44] should be performed for the further studies.

## 5. Conclusion

In conclusion, the alteration of network attributes for the whole and each subnetworks following the progression of AD was explored by the graph theory. For the whole-brain network analysis, the significant difference was found only between the NC and patient groups, while between different patient groups in AD progression, we discovered little significant difference. For the subnetwork analysis, two types of distinct change patterns, named ‘temperature inversion' and “monotonous decline,” were observed. We supposed that the work mechanisms of subnetworks with same evolving pattern may have something the same in nature. And the attributes of each subnetwork (the intra- and intersubnetwork functional connectivity) for each disease stage have varying degrees of dynamical changing, mainly involving DMN and visual networks. These results may shed lights on the pathophysiological mechanism of AD progression.

## Figures and Tables

**Table 1:** Demographic characteristics of the studied cohort.

	NC	EMCI	LMCI	AD
Number	35	37	33	25
Gender (M/F)	14/21	16/21	19/14	14/11
Age (year)	73.80 ± 5.06	72.96 ± 4.55	74.03 ± 4.65	75.17 ± 4.08
MMSE	28.89 ± 1.21	28.08 ± 1.74	27.85 ± 1.64	22.72 ± 2.41

**Table 2:** Introduction of graph theory properties^∗^.

Name	Abbreviation	Expression
Characteristic path length	Char	Char = 2/*N*(*N* − 1)∑_*i*>*j*_dis_*i*,*j*_
Efficiency	Effi	Effi = 1/*N*(*N* − 1)∑_*i*≠*j*_(1/dis_*i*,*j*_)
Clustering coefficient	Clus	Clus = 1/*N*∑*C*_*i*_ *C*_*i*_ = *t*_*i*_/*k*_*i*_(*k*_*i*_ − 1)/2
Transitivity	Tran	Tran = ∑_*i*_2*t*_*i*_/∑_*i*_*k*_*i*_(*k*_*i*_ − 1)
Small worldness	SW	SW = *C*/*C*_0_/*L*/*L*_0_

where *N* is the total number of nodes in the network, dis_*i*,*j*_ is the minimum number of hops from nodes numbered as *i* to nodes numbered as *j*, *k* is the degree of nodes numbered as *i*, that is, the number of neighbors, while *t*_*i*_ is the number of edges between neighbors of nodes numbered as *i*. *L*_0_ and *C*_0_ are the length of the characteristic path and clustering coefficient of random network under the same network size and density, respectively.

## Data Availability

The data used to support the findings of this study are available from the the Alzheimer's Disease Neuroimaging Initiative (ADNI) database (https://adni.loni.usc.edu) and corresponding author upon request.
